# Pediatric eMental healthcare technologies: a systematic review of implementation foci in research studies, and government and organizational documents

**DOI:** 10.1186/s13012-017-0608-6

**Published:** 2017-06-21

**Authors:** Nicole D. Gehring, Patrick McGrath, Lori Wozney, Amir Soleimani, Kathryn Bennett, Lisa Hartling, Anna Huguet, Michele P. Dyson, Amanda S. Newton

**Affiliations:** 1 Department of Pediatrics, Faculty of Medicine & Dentistry, University of Alberta, 11405-87 Avenue, Edmonton, AB T6G 1C9 Canada; 2 IWK Health Centre, 5850—5980 University Avenue, Halifax, NS Canada; 3 Centre for Research in Family Health, IWK Health Centre, Halifax, NS Canada; 4 Department of Health Research Methods, Evidence and Impact (Formally Clinical Epidemiology and Biostatistics), and Offord Centre for Child Studies, McMaster University, Hamilton, ON Canada; 5 Department of Community Health and Epidemiology, Dalhousie University, Halifax, NS Canada

**Keywords:** eHealth, Mental health, Implementation science, Healthcare planning, Organizational innovation, Decision-making, Healthcare organizations

## Abstract

**Background:**

Researchers, healthcare planners, and policymakers convey a sense of urgency in using eMental healthcare technologies to improve pediatric mental healthcare availability and access. Yet, different stakeholders may focus on different aspects of implementation. We conducted a systematic review to identify implementation foci in research studies and government/organizational documents for eMental healthcare technologies for pediatric mental healthcare.

**Methods:**

A search of eleven electronic databases and grey literature was conducted. We included research studies and documents from organization and government websites if the focus included eMental healthcare technology for children/adolescents (0–18 years), and implementation was studied and reported (research studies) or goals/recommendations regarding implementation were made (documents). We assessed study quality using the Mixed Methods Appraisal Tool and document quality using the Appraisal of Guidelines for Research & Evaluation II. Implementation information was grouped according to Proctor and colleagues’ implementation outcomes—acceptability, adoption, appropriateness, cost, feasibility, fidelity, penetration, and sustainability—and grouped separately for studies and documents.

**Results:**

Twenty research studies and nine government/organizational documents met eligibility criteria. These articles represented implementation of eMental healthcare technologies in the USA (14 studies), United Kingdom (2 documents, 3 studies), Canada (2 documents, 1 study), Australia (4 documents, 1 study), New Zealand (1 study), and the Netherlands (1 document). The quality of research studies was excellent (*n* = 11), good (*n* = 6), and poor (*n* = 1). These eMental health studies focused on the acceptability (70%, *n* = 14) and appropriateness (50%, *n* = 10) of eMental healthcare technologies to users and mental healthcare professionals. The quality of government and organizational documents was high (*n* = 2), medium (*n* = 6), and low (*n* = 1). These documents focused on cost (100%, *n* = 9), penetration (89%, *n* = 8), feasibility (78%, *n* = 7), and sustainability (67%, *n* = 6) of implementing eMental healthcare technology.

**Conclusion:**

To date, research studies have largely focused on acceptability and appropriateness, while government/organizational documents state goals and recommendations regarding costs, feasibility, and sustainability of eMental healthcare technologies. These differences suggest that the research evidence available for pediatric eMental healthcare technologies does not reflect the focus of governments and organizations. Partnerships between researchers, healthcare planners, and policymakers may help to align implementation research with policy development, decision-making, and funding foci.

**Electronic supplementary material:**

The online version of this article (doi:10.1186/s13012-017-0608-6) contains supplementary material, which is available to authorized users.

## Introduction

The global prevalence of mental disorders in children and adolescents is reported to be as high as 30% [1–4]. Under-diagnosis and under-treatment of childhood mental disorders are well-documented concerns [2, 5–7]. The current distribution, demand, structure, and costs that underpin pediatric mental healthcare services make them relatively unavailable to many of those who need them [8]. Electronic mental healthcare (eMental healthcare) technologies, which broadly include Internet-, mobile-, and, computer-based programs and resources as well as mobile phone applications, are considered promising approaches to enable more efficient use of mental healthcare resources, lower access barriers to traditional face-to-face mental healthcare, and provide flexibility in terms of standardization and personalization, interactivity, and consumer engagement [9–14].

Researchers (e.g., those developing and/or evaluating eMental healthcare technologies), healthcare planners (e.g., administrators in agencies either using or desiring to use eMental healthcare technologies), and policymakers (e.g., individuals with authority to set eMental healthcare policy for a healthcare organization or system) all convey interest and a sense of urgency in using eMental healthcare technologies to improve pediatric mental healthcare availability and access. To date, these three stakeholder groups have focused on different aspects of implementation. Researchers have studied user satisfaction [13, 15, 16] to determine that eMental healthcare technologies are acceptable to children and adolescents, and their parents and mental healthcare professionals. Healthcare planners and policymakers have discussed issues such as the cost and feasibility of eMental healthcare technologies to deliver pediatric mental healthcare [17]. Current priorities that are relevant to all three stakeholders groups are generating evidence to demonstrate how eMental healthcare technologies can be optimally incorporated within an existing healthcare system and how technology implementation can be supported within an organization or system (e.g., governance, policy, funding) [18–20]. These priorities may be optimally achieved through collaborations between researchers, healthcare planners, and policymakers that aim to generate evidence for integrating and supporting eMental healthcare technologies in healthcare systems. To provide recommendations for such collaborations, we conducted a systematic review to identify what aspects of implementation have been studied and reported on for pediatric eMental healthcare technologies and what implementation goals/recommendations are present in government and organizational documents relating to eMental healthcare technologies for pediatric mental healthcare. Our aim was to identify and compare current areas of focus among research studies and government/organizational documents and to use these areas of focus to propose recommendations for implementation research, policies, and funding.

## Methods

### Design

We systematically reviewed the literature to identify research studies and government and organizational documents with information regarding the implementation of eMental healthcare technologies for children and adolescents. We used a protocol that was developed a priori to define the objective, outline the search strategy, establish selection (inclusion/exclusion) criteria, determine implementation findings, guide the data collection process, and define the analysis. We followed the PRISMA statement checklist for reporting [21].

### Search strategy

A research librarian developed and implemented the systematic search strategies using language (English) restrictions. The search was conducted in 11 electronic bibliographic databases: Medline, CINAHL, Embase, EBM Reviews, ProQuest Theses and Dissertations, Ovid MEDLINE In-Process & Other Non-Indexed Citations, OVID HealthStar, Cochrane Database of Systematic Reviews, Health Technology Assessment Database, ACP Journal Club, and SocIndex. The final Medline strategy is provided (see Additional file 1). Search terms focused on population and technology parameters used to screen for study eligibility. We also included terms related to “attitudes,” “preferences,” and “diffusion of innovation.” Thus, the search strategy was broad in order to identify potentially eligible studies that may not have been indexed using specific implementation science terms. The search was executed in each database from inception to September 30, 2015. Although the search was executed from inception to September 30, 2015, we restricted inclusion to studies and documents published after 2005.

To identify unpublished research and research-in-progress, we searched Google, the U.S. National Institutes of Health Clinical Trials database, the Australian New Zealand Clinical Trials Registry, the International Clinical Trials Registry Platform, and the UK Clinical Trials Gateway. To identify government and organizational documents, we conducted a two-pronged search: (1) targeted Google searches for relevant government, health, and technology organizations having clearly stated goals and/or funding relating to eHealth and behavioural technologies and (2) recommendations from members of our team. Overall, we created a list of 38 government and organizational websites, which was reviewed by the research team (see Additional file 2 for the full list). We also sought documents from key contacts responsible for leadership, policy, research, and information technology considered to be influential in the use of eHealth technologies. These contacts represented Canada’s Mental Health Commission e-mental health steering committee and the Ontario Centre of Excellence for Child and Youth Mental Health, the Australian Government’s Mental Health Commission and Department of Health and Ageing e-mental health expert advisory committee, the Netherland’s Dutch Association of Mental Health and Addiction Care, New Zealand’s National Health IT Board, and United Kingdom’s National Collaborating Centre for Mental Health and Mental Health Network. Reference lists of included studies and documents were also searched.

### Criteria for including studies and documents for this review

We restricted the study and government/organizational document inclusion to countries from the largest English speaking eHealth markets [22]. These countries were Australia, Canada, the Netherlands, New Zealand, the United Kingdom, and the USA. Studies of any design were eligible for inclusion.

### Population of interest

Studies and government/organizational documents that included children and/or adolescents (0–18 years) as participants (studies only) or a population of interest (documents) were considered for inclusion. Government and organizational documents that focused on eMental healthcare technologies for all ages (including children and/or adolescents) were also eligible for inclusion.

### eMental healthcare technology

Studies and government/organizational documents were eligible for inclusion if they evaluated/focused on eMental healthcare technology that met our definition: Internet-, computer-, or mobile-based programs and applications (‘apps’). Studies and documents of eMental healthcare technologies focused exclusively on phone calls or teleconferencing were excluded from the review as these technologies did not meet our definition of eMental healthcare. Studies and documents that focused on eMental healthcare technology use with parents of children with a mental health need or pediatric healthcare professionals were eligible for inclusion.

### Implementation findings, goals, and/or recommendations

All outcomes, goals, and recommendations relating to implementation were considered. We used Proctor and colleagues’ eight outcome categories for implementation research [23] as a framework to identify outcomes, objectives, goals, and recommendations of interest in the studies and documents. To be included, a study needed to evaluate and report on at least one of the eight categories, and a document needed to contain at least one goal and/or recommendation that related to a category. The eight categories are as follows: acceptability, adoption, appropriateness, cost, feasibility, fidelity, penetration, and sustainability [23]. These outcomes have shaped traditional mental health services integration in routine care [24–28] and provide a common taxonomy for examining eMental healthcare technology implementation. For the purpose of this review, the categories were defined as follows: *acceptability*, a measure of satisfaction with the technology (including attitudes, functionality, preferences, and user experience); *adoption*, the intention, initial decision, or action to try or employ technology (e.g., uptake and utilization); *appropriateness*, the perceived fit, relevance, usefulness/helpfulness, or compatibility of the technology for a given practice setting, professional, or user and/or perceived fit of a technology to address a particular issue or problem; *cost*, the financial impact of an implementation effort (including measures of cost-effectiveness or cost-benefit); *feasibility*, the extent to which a technology could be successfully used or carried out within a setting (including utility, compatibility, and barriers); *fidelity*, the degree to which a technology was implemented as it was intended such as adherence; *penetration*, the integration of a practice within a service setting and its subsystems (e.g., “spread” or “reach”); and *sustainability*, the extent to which a newly implemented treatment is maintained or integrated within a service setting’s ongoing, stable operations [23].

### Screening for eligibility

Studies and documents were organized and screened using EndNote X7.2.1. Studies and documents were first screened at the title and abstract level (stage 1 screening) to determine whether they met the inclusion criteria. At stage 1, two reviewers (NDG, AS) independently screened the title and abstract for the first 100 studies/documents in the library and subsequently calculated inter-rater agreement with the kappa statistic [29]. The agreement was not sufficiently high (Cohen’s kappa, *κ* = 0.70), and we determined that “implementation” was an unclear term to aid in determining whether studies and documents provided implementation information on implementation outcomes, objectives, goals, and/or recommendations. We introduced Procter’s implementation outcomes framework [23] to the review protocol at this time, and two reviewers independently screened another 100 studies/documents in the library. This screening resulted in “almost perfect agreement” (Cohen’s kappa, *κ* = 0.84) [30], which indicated consensus on the definition of implementation. The remaining studies/documents in the library were then divided in two with each reviewer taking a respective half to screen using the title and abstract. Any studies/documents where it could not be determined whether they met inclusion criteria using the article’s title and abstract progressed to a review of the full-text (stage 2 screening) to determine eligibility. Any discrepancies were discussed between the reviewers and taken to a third party (ASN) if no agreement could be reached.

### Data extraction

Data were extracted into a standardized form (Microsoft Excel; Microsoft, Redmond, Washington, USA). Extracted data were: (1) key article characteristics (e.g., author, date of publication, country); (2) study or document objectives; (3) technology type(s) (Internet-, mobile-, or computer-based) and services/treatments delivered; (4) details about research study design; (5) target population/study participants; and (6) implementation outcomes and findings from studies and implementation goals and/or recommendations from government/organizational documents according to Proctor et al. (acceptability, adoption, appropriateness, cost, feasibility, fidelity, penetration, and sustainability) [23]. Included studies and documents were divided between two reviewers (NDG, AS) who extracted the data from their respective half and then checked the other’s extraction for accuracy and completeness. Discrepancies were resolved by discussion and/or by contacting corresponding authors of included studies/documents for clarification.

### Quality assessment

The quality of the research studies was assessed using the Mixed Methods Appraisal Tool (MMAT) [31]. The MMAT is applicable to quantitative, qualitative, and mixed methods studies. The scoring scale ranges from 0 (low quality) to 100 (high quality) and has been pilot tested for reliability in systematic reviews [32]. Ratings are specific to particular methodologies and are based on control of confounding factors, completeness of outcome data, minimization of selection bias, representativeness of sample, appropriateness of measures, response and withdrawal rates, appropriateness of study design to answer the research questions, and consideration of limitations. Two reviewers (NDG, AS) independently completed the MMAT for each included study and inter-rater agreement was considered “almost perfect” (Cohen’s kappa, *κ* = 0.81) [30]. Discrepancies were resolved by a third party (ASN).

The quality of government and organizational documents was assessed using the Appraisal of Guidelines for Research & Evaluation II (AGREE II) [33]. AGREE II assesses six domains: scope and purpose, stakeholder involvement, rigor of development, clarity of presentation, applicability, and editorial independence. While originally intended for clinical practice guidelines, most domains are applicable to government and organizational documents. Two domains, “clarity of presentation” and “applicability,” have criteria specific to guideline recommendations; however, because we were interested in document recommendations, these domains were applicable to our use. The AGREE II provided an overall quality score from 0% (low quality) to 100% (high quality), and a recommendation as to whether the document was recommended: (1) for use, (2) for use with modifications, or (3) not for use. Two reviewers (NDG, AS) independently completed the AGREE II for each document. Domain and overall quality scores were calculated by summing up the scores given by two reviewers [34]. Recommendations for document use were decided by consensus between the two reviewers. Discrepancies in recommendations for document use were resolved by a third party (ASN).

### Data analysis

Data analysis followed two approaches. First, two reviewers (NDG, AS) conducted a narrative (descriptive) synthesis [35] to produce a summary of the research studies and government/organizational documents included in the review. This summary encompassed the five domains for which we extracted data. Second, implementation data from each research study and government/organizational document were grouped according to the eight implementation outcomes framework [23]. A cell remained empty if there was no relevant data pertaining to an implementation outcome. Data categorization was reviewed and discussed by NDG, ASN, and LW until consensus was achieved, and all data were coded into appropriate outcomes.

As a final step, a template approach to text analysis [36] was undertaken by two reviewers (NDG, AS). This approach involved bringing together the narrative synthesis and grouped implementation data under the implementation outcomes framework. This analytic step allowed the research team to identify the implementation foci of research studies as compared to government/organizational documents so that recommendations for implementation research, policies, and funding could be formulated.

## Results

### Literature search and selection

**Fig. 1:**
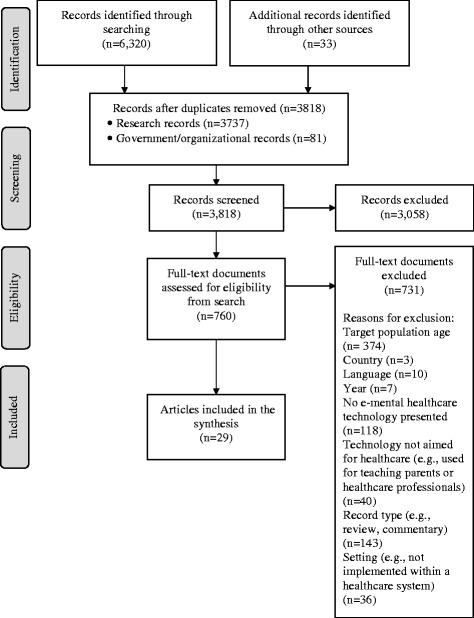
Literature search flow diagram

As shown in Fig. 1, after the removal of duplicates, the literature search identified 3818 articles for screening; 3737 research articles and 81 government and organizational documents. A total of 3058 articles were excluded after screening the titles and abstracts. The full texts of the remaining 760 articles were reviewed. Of these, 29 articles were included in the review: 9 government/organizational documents and 20 research studies.

### Research study and government/organizational document characteristics

As shown in Table 1, of the nine government and organizational documents, five focused primarily on eMental healthcare services for youth [17, 37–40] while the remaining discussed eMental healthcare for the general population, including youth [41–43]. Although some reports contained research elements (i.e., literature reviews, stakeholder interviews, surveys) [38–40, 42, 44], they were categorized as government/organizational documents given their affiliations. Four documents originated from Australia [37, 38, 43, 44], two from the United Kingdom [39, 42], two from Canada [17, 40], and one from the Netherlands [41]. Following quality assessment, one document was found to be of poor quality (25%) and was not to be recommended for use [41], six documents were found to be of medium quality (range 58–83%) and were recommended for use given modification [17, 37–39, 42, 44], and two documents were found to be of high quality (92%) and were recommended for use without modifications [40, 43] (see Additional file 3).

**Table 1:** Government and organizational reports on eMental healthcare implementation

Report (year, country)	Objectives	Target audience	Implementation focus	
			General population	Children and adolescents
*A Way Forward: Equipping Australia’s Mental Health System for the Next Generation* (2015, AUS) [37]	1. To examine the current and future states of mental health and mental health service provision in Australia, specifically in terms of cost-effectiveness	- Mental Health Commission	✓	✓
*E-Mental Health Services in Australia 2014: Current and Future* (2014, AUS) [43]	1. To propose achievable and scalable solutions to unlock the even greater potential of eMental health for the community	- Government - Mental Health Commission	✓	
*Strategies for Adopting and Strengthening E-mental Health* (2014, AUS) [38]	1. To identify the benefits, issues and obstacles for the provision of online health services in the mental healthcare system 2. To determine what investment and development, including infrastructure and processes, is required for improved integration of evidence-based online mental health services	- Mental Health Commission	✓	✓
*E-therapies Systematic Review for Children and Young People with Mental Health Problems* (2014, UK) [39]	1. To determine the availability of computer-based applications on the Internet for children and young people with mental health problems 2. To determine the acceptability of programs and to investigate aspects of concern and value to young people	- Government	✓	✓
*The Future’s Digital: Mental Health and Technology* (2014, UK) [42]	1. To examine the case for digital transformation 2. To determine how digital technology is currently being used in the design and delivery of mental health services 3. To determine what actions national bodies and individual professionals, as well as commissioners, take to ensure digital technology is leveraged to its full potential	- Government - Decision-/policymakers - Healthcare professionals	✓	
*E-mental Health in Canada: Transforming the Mental Health System using Technology* (2014, CAN) [17]	1. To examine the spectrum of current eMental health technology/tools 2. To determine the key considerations examining the transformational potential of and barriers to the use of eMental health 3. To examine national and international eMental health approaches, including promising-practices 4. To provide recommendations for the future of eMental health	- Government - Decision-/policymakers - Healthcare professionals - Funders (i.e., insurers) - Academics - eMental healthcare technology developers	✓	✓
*E-mental Health in the Netherlands* (2013, NED) [41]	1. To showcase how eMental Health utilizes technological developments to respond to today’s challenges, while at the same time increasing the number of people in reach of mental healthcare, thus decreasing the treatment gap	- Individuals at all levels in the Netherlands	✓	
Using Technology to Deliver Mental Health Services to Children and Youth in Ontario (2013, CAN) [40]	1. To engage with policy- and decision-makers to identify their perspectives on eMental Health 2. To conduct a review of the literature on eMental Health 3. To provide recommendations	- Government - Decision-/policymakers - Healthcare professionals	✓	✓
*The eHealth Readiness of Australia’s Medical Specialists* (2011, AUS) [44]	1. To explore whether Australian medical specialists are ready to adopt and use eHealth technologies and solutions today and in a way consistent with policy direction in the future 2. To identify the barriers impacting eHealth readiness and adoption and explore how to minimize them 3. To identify eHealth enablers and explore how to apply them to drive adoption and effective usage	- Government - Healthcare professionals	✓	

Table 2 outlines the 20 research studies included in the review. Fourteen studies were from the USA [45–58], three from the United Kingdom [59–61], and one each from Australia [62], Canada [63], and New Zealand [64]. Across the studies, fourteen examined eMental healthcare technologies to be used by children and adolescents [45, 46, 48–51, 54–56, 59–62, 64], three examined technologies to be used by healthcare professionals when interacting with pediatric patients [47, 53, 63], and three examined technologies to be used by parents on behalf of their child [52, 57, 58]. One study was assessed as being of extremely poor quality, receiving a MMAT score of 0 [50], two studies were of poor quality and received a score of 50 [48, 49], six were of good quality and received a score of 75 [47, 51, 53, 56, 58, 61], and the remaining 11 were of excellent quality with a score of 100 [45, 46, 52, 54, 55, 57, 59, 60, 62–64]. An additional file shows details of the quality assessment (see Additional file 4).

**Table 2:** Research studies that have examined the implementation of eMental healthcare technologies, listed in order of publication date

Author (year, country)	Technology	Implementation of technology	Participants and setting	Individuals studied as part of implementation evaluation				
				Children/adolescents	Parents	Healthcare professionals	Healthcare planners	Healthcare policymakers
Hetrick et al. (2015, AUS) [62]	Online monitoring tool of depressive symptoms, suicidality, and side effects (via iPad).	Adolescents completed the tool once a week for up to 3 months. They could fill in the tool at any location with Internet access at any time with the exception of suicidal ideation items; these items were completed at the beginning of their regular treatment session with their clinician on an iPad. Clinicians received a chart of scores after 4 weeks so that they could share with their patient and received an email regarding side effects that were endorsed for immediate action.	Adolescents, aged 14–24 years, receiving mental healthcare at the Youth Mood Clinic (YMC) Mental health clinic	✓		✓		
Reuland et al. (2014, USA) [48]	CBM-I (Cognitive Bias Modification for Interpretation)	Online intervention where adolescents were instructed to read and imagine themselves in 50 scenarios per session that were ambiguous in meaning until a word fragment near the end of the scenario resolved the ambiguity in a positive way (e.g., in a way inconsistent with socially anxious beliefs)	Socially anxious adolescents, aged 10-15 years, and their mothers No specific setting	✓	✓			
Gonzales et al. (2014, USA) [45]	Text message^a^		Young people, aged 12–24 years, receiving outpatient or residential substance abuse treatment Outpatient and residential substance abuse treatment programs	✓				
Gladstone et al. (2014, USA) [49]	CATCH-IT: Competent Adulthood Transition with Cognitive-behavioural Humanistic and Interpersonal Training	14 online modules of Internet training to teach adolescents how to reduce behaviors that increase depressive disorders. Modules use CBT, behavioral activation, interpersonal psychotherapy, and community resiliency concept model.	Young people, aged 14–21 years, with a general primary care concern Primary care settings	✓				
Eisen et al. (2013, USA) [50]			Primary care professionals and young people, aged 14–21 years, with a general primary care concern Primary care settings	✓		✓		
Iloabachie et al. (2011, USA) [51]			Young people, aged 14–21 years, with a general primary care concern who had positive screens for sub-threshold depressive symptoms, and parents of those who were <18 years Primary care settings	✓	✓			
Fothergill et al. (2013, USA) [52]	Online screener (via computer or tablet)	25 questions, that can branch into as many as 57 questions based on responses, regarding somatic and mental health concerns, general health risk, anxiety, and parental depression. The screener calculates scores for the validated scales it contains. A summary screen tallies the positive responses within broad categories and highlights scores for the validated assessments above the standard cut-offs	Primary care professionals and parents presenting for a well child visit Primary care settings		✓	✓		
Branson et al. (2013, USA) [46]	Text message	Reminders sent the evening before each scheduled therapy session (e.g., “C u Wed @8”)	Adolescents, aged 13–17 years Hospital-based outpatient mental health clinic	✓				
Han et al. (2013, USA) [53]	Toolkit on the MDPC Website	Health questionnaire (PHQ-9), education material for patients, guides to diagnostic and treatment approaches, specialty care referral forms, slide presentation, training manuals, publications, cost calculator (investment savings for employers)	Healthcare professionals using the MDPC website			✓		
Salloum et al. (2013, USA) [56]	Camp Cope-A-Lot: cCBT program for childhood anxiety within community mental health centers	Therapist provides monitoring and coaching as the child completes the program. The therapist is present during program completion to answer any questions and build therapeutic alliance. 12 weekly sessions: sessions 1 to 6 focus on skill-building and sessions 6 to 12 are exposure-based sessions where the therapist provides direct coaching	Children aged 7–13 years, with an anxiety disorder, their parents, administrators, study therapists Community-based mental healthcare settings	✓	✓	✓	✓	
Merry et al. (2012, NZ) [64]	SPARX (Smart, Positive, Active, Realistic, X-factor thoughts)	Interactive fantasy game designed to deliver cognitive behavioural therapy for the treatment of clinical depression. 7 modules delivered over a period of 4 to 7 weeks. A “guide” puts the game into context, provides education, gauges mood, and sets and monitors real-life challenges	Adolescents, aged 12–19 years, seeking help for mild to moderate depressive symptoms that were assessed by a clinician as being fit for self-help and not being a high risk for suicide or self-harm Primary care settings	✓				
Ahmad et al. (2012, CAN) [63]	Computer-assisted interactive health risk assessment tool	A health risk assessment tool that provides feedback to both the patient and the physician on psychosocial health. The tool considers the contextual details of the patient (e.g., violence, drug or alcohol abuse)	Healthcare professionals (nurses, physicians, social workers, etc.) Primary and acute care settings			✓		
Murphy et al. (2011, USA) [57]	Electronic Outcomes Rating Form (e-ORF) in conjunction with a web-based patient tracking system	The e-ORF is an electronic form filled out by parents of all intake patients using a digital pen. The form includes assessment tools (BPRS-C, CGAS). The e-ORF automatically prints outcome forms of routine paperwork for intake visit and follow-up forms every 90 days to reduce burden on the administrative and clinical staff. The digital pens have the ability to enter the assessment data directly into the hospital’s database	Children and adolescents ≤18 years undergoing outpatient mental health evaluation Outpatient child psychiatric clinic		✓			
Diamond et al. (2010, USA) [54]	BHS (Behavioural Health Screen)	Screening tool assesses risk behaviors and psychiatric symptoms in 13 modules. Patient completes the BHS in a waiting room, the report printed at primary care office, and the summary of assessment given to physician	Adolescents with a general primary care concern Primary care settings	✓				
Fein et al. (2010, USA) [55]	BHS-ED (Behavioural Health Screen–Emergency Department)	Psychosocial assessment tool designed for adolescents in non-psychiatric medical settings. Nurses or medical technicians logged the patient onto the website and registered them with a password and medical record number. The BHS-ED began with a slide and audio show that explained the rationale for the screening and the standard limits of confidentiality	Adolescents, aged 14–18 years, without acute or critical injuries or illness, presenting with non-psychiatric symptoms Emergency department of an urban tertiary care children’s hospital	✓				
Stallard et al. (2010, UK) [61]	cCBT	Focus was whether mental health professionals would consider the delivery of CBT via computer technology	Mental health professionals National conference, British Association of Behavioral and Cognitive Psychotherapy			✓		
Pretorious et al. (2010, UK) [59]	Web-based CBT for bulimic disorders	8 interactive, multimedia sessions, electronic message board for participants and parents, and email support provided by therapist (flexible weekly support and advice via email)	Young women, aged 16–20 years, with bulimia nervosa or atypical bulimia nervosa Clinic	✓				
Horwitz et al. (2008, USA) [58]	CHADIS (Child Health and Development Interactive System)	CHADIS provides access to 23 different questionnaires and asks parents prioritize their concerns so clinicians can plan agenda for the upcoming appointment	Parents of children <8 years presenting for a well-child visit and pediatricians Primary care and community-based mental healthcare settings		✓	✓		
John et al. (2007, USA) [47]	Personal digital assistant (PDA) decision support system (DSS)	Screening questions supporting the PDA application: Short Mood and Feeling Questionnaire (SMFQ) and four additional questions, two related to family history of depression and two related to suicide	Pediatric Advanced Practice Nursing students treating children aged 8 to 18 years University-based medical centre			✓		
Hanley et al. (2006, UK) [60]	Online counseling services for youth^a^		Counselors Online forum			✓		

*BPRS-C* Brief Psychiatric Rating Scale for Children, *CBT* cognitive behavioral therapy, *CGAS* Children’s Global Assessment Scale, *PHQ-9* Patient Health Questionnaire, *SPARX* Smart, Positive, Active, Realistic, X-factor thoughts, *cCBT* computerized cognitive behavioral therapy, *NR* Not reported

^a^Intervention features are not reported as the study focused on identifying features to develop the intervention

### Implementation outcomes

Table 3 presents the implementation outcomes and findings from research studies and implementation goals and recommendations from government and organizational documents, organized according to Proctor and colleagues’ implementation outcomes.

**Table 3:** Implementation outcomes investigated by research studies and addressed/recommended in government and organizational documents

Author (year, country)	Implementation outcome investigated							
	Acceptability	Adoption	Appropriateness	Cost	Feasibility	Fidelity	Penetration	Sustainability
Research studies								
Hetrick et al. (2015, AUS) [62]	✓		✓					
Gonzales et al. (2014, USA) [45]	✓							
Reuland et al. (2014, USA) [48]	✓				✓	✓		
Gladstone et al. (2014, USA) [49]	✓							
Eisen et al. (2013, USA) [50]	✓		✓					
Fothergill et al. (2013, USA) [52]	✓		✓					
Han et al. (2013, USA) [53]	✓	✓						
Salloum et al. (2013, USA) [56]	✓			✓				
Branson et al. (2013, USA) [46]	✓	✓	✓					
Ahmad et al. (2012, CAN) [63]			✓		✓			
Merry et al. (2012, NZ) [64]	✓		✓			✓		
Murphy et al. (2011, USA) [57]		✓				✓		
Iloabachie et al. (2011, USA) [51]	✓							
Diamond et al. (2010, USA) [54]	✓							
Fein et al. (2010, USA) [55]		✓						
Pretorious et al. (2010, UK) [59]	✓		✓					
Stallard et al. (2010, UK) [61]			✓					
Horwitz et al. (2008, USA) [58]	✓							
John et al. (2007, USA) [47]		✓	✓					
Hanley et al. (2006, UK) [60]			✓					
	Implementation outcome addressed/recommended							
Government and organizational documents								
Reach Out (2015, AUS) [37]	✓			✓	✓		✓	
eMHA (2014, AUS) [43]	✓			✓	✓	✓	✓	✓
NCCMH (2014, UK) [39]	✓			✓		✓	✓	
MHCC (2014, CAN) [17]	✓		✓	✓	✓	✓	✓	✓
Sax (2014, AUS) [38]			✓	✓	✓	✓	✓	✓
MHN (2014, UK) [42]			✓	✓	✓		✓	✓
OCE (2013, CAN) [40]			✓	✓	✓	✓	✓	✓
GGZ (2013, AUS) [41]				✓		✓		
DHA (2011, AUS) [44]	✓		✓	✓	✓	✓	✓	✓

*OCE* Ontario Centre of Excellence, *eMHA* e-Mental Health Alliance, *NCCMH* National Collaborating Centre for Mental Health, *MHCC* Mental Health Commission of Canada, *MHN* Mental Health Network, *GGZ* Geestelijke gezondheidszorg, *DHA* Department of Health and Ageing

### Acceptability

Fourteen research studies (70%) examined acceptability (ten quantitatively [46, 49–54, 56, 58, 64], three [48, 59, 62] qualitatively, and one using both qualitative and quantitative methods [45]). The majority of studies were of good or excellent quality [45, 46, 51–54, 56, 58, 59, 62, 64]; three were of poor quality [48–50]. How acceptability was defined varied considerably across studies: satisfaction with the technology [46, 56, 64], functionality of the technology [62], attitudes towards the technology [50], technology preferences [51, 59], user experience using the technology [52, 56, 58, 59], and acceptability of the technology [45, 48, 54]. Studies of acceptability reported that participants responded favorably to eMental healthcare technologies [45, 46, 48–50, 53, 54, 58, 62, 64] and liked that they provided autonomy, convenience, anonymity, and accessibility [51, 52, 56, 59, 62, 64]—particularly when technologies were designed specifically for the youth [54, 56, 64].

Five government/organizational documents (56%) included goals or recommendations relating to the acceptability of eMental healthcare technology. Documents were mainly of medium quality (range 58–83%) [17, 37, 39, 44]; although, one document was of high quality [43]. These documents described the need for better incorporation of consumer preference during design planning to ensure such services are user-centered and individualized [17, 39, 43, 44] and emphasized the need to increase public awareness of the acceptability of eMental healthcare technology [17, 37, 43].

### Adoption

Five studies (25%), of good or excellent quality, measured adoption of eMental health technology for pediatric mental healthcare [46, 47, 53, 55, 57]: pre-post measurements of treatment attendance [46], screening rates [47, 55], number of intake appointments [57], and uptake into healthcare practice [53]. Studies showed that children and adolescents receiving the eMental healthcare technology demonstrated significantly higher rates of attendance [46], and electronic technologies were associated with improved clinician completion rates [57]; however, studies concerned with healthcare professional adoption showed moderate screening rates and uptake into practice [47, 53, 55]. Government and organizational documents did not address actual eMental healthcare technology adoption; although, documents did discuss the intent to use technology to provide mental healthcare.

### Appropriateness

Appropriateness was examined in ten studies (50%) [46, 47, 50, 52, 59–64]. One study was of poor quality [50], and the remaining were of good or excellent quality [46, 47, 52, 59–64]. The definition of appropriateness varied considerably across studies: appropriateness [46, 47, 50, 52, 60, 61, 63], relevance [59, 63], usefulness [59, 61–64], suitability [59, 61, 64], and perceived fit [64]. Of these studies, five measured this construct qualitatively [47, 52, 59, 60, 62], four quantitatively [46, 50, 63, 64], and one using both qualitative and quantitative measures [61]. Most studies examined the appropriateness of eMental healthcare technology for healthcare professionals and their settings [46, 47, 50, 52, 60–63], while some studies examined the appropriateness of the technology for children and adolescents [59, 61, 64]. Computer-based treatments and online management systems were deemed appropriate to mental healthcare practices in that the technologies allowed for less preparation time and provided facilitation and appointment planning [52, 62]. Adolescents found eMental healthcare helpful for improving appointment attendance [46], and one study demonstrated healthcare professional competency and compatibility at employing the technology within practice [50]. Healthcare professionals regarded a tool’s use and appropriateness as very important for successful implementation [63] and believed eMental healthcare had the potential to be helpful for children and adolescents, especially for mild to moderate problems [61]. However, some studies showed that healthcare professionals perceived interference with the therapeutic relationship due to eMental healthcare technologies [47, 60]. Similarly to acceptability, the accessibility, flexibility, and anonymity of eMental healthcare were factors that influenced treatment preference for web-based over face-to-face interventions [59] and eMental healthcare was at least as good a treatment as usual in primary healthcare sites [64].

Five government and organizational documents (56%) addressed appropriateness. One document was of high quality [40], while the remaining were of medium quality (range 58–83%) [17, 38, 42, 44]. Documents considered appropriateness from the perspectives that (1) additional research and development should be conducted to assure healthcare quality and safety standards are not compromised [17, 40, 42, 44], (2) new eMental healthcare technologies should be integrated and evaluated within existing health and technology policies (including IT aspects) [17, 40, 42, 44], and (3) technologies need to be accessible to rural, regional, and indigenous communities [38, 40] to ensure appropriateness to these populations.

### Cost

Of the 20 research studies, one qualitative study (5%) of good quality considered cost issues from system and organization perspectives and described the need to address cost coverage in terms of who covers treatment costs and how to integrate third party payers [56].

All nine government and organizational documents (100%) cited cost as a major component of future eMental healthcare implementation efforts [17, 37–44]. One of these documents one was of low quality [41], four were of medium quality (range 58–83%) [37, 38, 42, 44], and one was of excellent quality [40]. Across the government and organizational documents, start-up costs for developing or implementing technology into practice [17, 37, 38, 40], reimbursement to consumers from health insurance companies [17, 39, 44], the cost-effectiveness of blended care models [41], and billing requirements for healthcare professionals providing eMental healthcare services were discussed [17, 39, 44]. Overall, the government/organizational literature recommended establishing and evaluating a sustainable funding model to address high development and continuing maintenance costs [17, 37, 39, 42–44] and allocating more government funds to further support research and development [42].

### Feasibility

Two studies (10%) quantitatively measured eMental healthcare technology feasibility [48, 63]. These studies were of poor quality [48] and excellent quality [63]. The definition of feasibility did not vary between the studies. While one of the studies found eMental healthcare technology to be feasible [48], the other found that healthcare professionals perceived professional development and workload as feasibility challenges (for example, rate changes to workload may be required when adopting an eMental healthcare technology within a professional’s clinical workflow) [63].

Seven government/organizational documents (78%) of excellent [40, 43] and medium quality (range 58–83%) [17, 37, 38, 42, 44] described the need to address feasibility if implementation of eMental healthcare technologies are to be successful [17, 37, 38, 40, 42–44]. Document recommendations included ensuring training and education programs for healthcare professionals and organizations as a means to ensure technology feasibility. That is, although a technology may be appropriate for a given setting, it may not be feasible to implement it if resources and training are not available [17, 37, 38, 40, 42–44].

### Fidelity

Three studies (15%) examined fidelity by quantifying adherence to the technology [48, 57] and reasons for non-completion [64]. Two of these studies were of excellent quality [57, 64] and one was of poor quality [48]. Studies reporting on fidelity found either perfect [48] or good [57, 64] adherence to the technology protocol, finding reasons for non-completion such as technical glitches and lack of time and/or interest [64].

Recommendations from seven government and organizational documents (67%) concentrated on the importance of fidelity in implementation evaluations [17, 38–41, 43, 44]. Of these documents one was of poor quality [41], four were of medium quality (range 58–83%) [17, 38, 39, 44], and two were of excellent quality [40, 43]. Government and organizational documents also described concerns about dropout and lack of follow-up with the use of eMental healthcare technologies [17, 38], and the need for evaluations to determine if such technologies are being delivered as intended and are as innovative as described [17, 38–41, 43, 44].

### Penetration and sustainability

Penetration and sustainability of eMental healthcare technologies were not considered in the research studies included in this review, but were addressed in government and organizational documents. Eight documents (89%) described recommendations related to the penetration of eMental healthcare technologies [17, 37–40, 42–44], while six described sustainability recommendations [17, 38, 40, 42–44]. Six of the documents recommending penetration outcomes were of medium quality (range 58–83%) [17, 37–39, 42, 44] and two were of excellent quality [40, 43]. Four documents examining sustainability outcomes were of medium quality (range 58–83%) [17, 38, 42, 44], and two were of excellent quality [40, 43]. Penetration recommendations included linking traditional and eMental healthcare services together at multiple points using a stepped care model to allow for cross-referral [17, 37–40, 42, 43] and conducting further research, development, and evaluation in routine clinical settings [39, 44] with large sample sizes to ensure scalability [17]. Sustainability recommendations included the need for policy reform by way of establishing standards for privacy and security [17, 40, 42, 44], devising a national eMental healthcare strategy/protocol and creating stricter governance to facilitate technology implementation [38, 42, 43], and using technology to foster collaboration [17].

## Discussion

There is an increasing interest and sense of urgency from the perspectives of researchers, healthcare planners, and policymakers to use eMental healthcare technologies in pediatric mental healthcare to improve healthcare availability and access. To date, however, these stakeholders have tended to focus on different aspects of implementation. If effective eMental healthcare services are to become a core component of routine service delivery, these different areas of focus need to be identified so that alignment of research, policies, and funding can occur. We undertook a systematic review to identify research studies and government and organizational documents that describe eMental healthcare technology implementation in healthcare systems for children and adolescents and explore what areas of implementation researchers, healthcare planners, and policymakers have historically focused on. This approach allowed us to identify areas of focus in the current eMental healthcare landscape in terms of implementation outcomes among different stakeholder groups and to use these areas of focus to propose recommendations for implementation research, policies, and funding. The takeaway points of this review are as follows:

### Implementation foci differ between researchers, healthcare planners, and policymakers

We found multiple differences in implementation outcome foci between government and organizational literature and research studies. Consistent with other reviews [18, 65], the research studies in this review predominantly focused on patient and clinician outcomes (e.g., use, satisfaction, acceptability). Areas of focus in government and organizational reports were cost, penetration, and feasibility. Differences in underlying positions between stakeholder groups may lead to opposing criteria for successful eMental healthcare implementation and/or may jeopardize stakeholder engagement [66]. The task of disseminating evidence on the success of these initiatives does not occur in separate asocial and apolitical bubbles [67]. They are often produced by, and in turn feed back into, the political process of deciding priorities and allocating resources to pursue them.

Distinct knowledge holders often have differing mentalities; governing bodies are often highly incentivized by the lowest cost for the most efficient use of resources, while research tends to be incentivized by innovation and/or validating their work [68, 69]. Thus, the differences in underlying priorities may reflect different values and goals of governing bodies and researchers. For example, due to underlying incentives, government and organization priorities may naturally gravitate towards long-term benefits of eMental healthcare (i.e., penetration and sustainability) as this aligns with the mandate of cost versus benefits. Given that eMental healthcare is a relatively new field, research may be appropriately focused on establishing an eMental healthcare innovation as acceptable and appropriate to users before studying other implementation constructs. As the field matures, there is an opportunity to study implementation outcomes, such as cost, penetration, and feasibility, that would be of interest and value to governments and organizations [23, 70]. Moving forward, alignment of funding interests and partnerships between stakeholder groups may allow for the development of funding models to study outcomes that require longer-term investigation and substantial effort such as fidelity, penetration, and sustainability. Future research endeavors may also benefit from using a structured, theory-driven methodology to compile, evaluate, and integrate eMental healthcare information such as the Health Information Technologies—Academic and Commercial Evaluation (HIT-ACE) methodology [71].

### A taxonomy for implementation outcomes is needed for the eMental healthcare technology field

Consistent with the broader literature, this review reflects a nonstandard usage of terminology and lack of consensus on a taxonomy relating to implementation metrics [72–74]. For example, there was overlap and inconsistency in the operationalization and discussion of appropriateness and acceptability; adoption was often used interchangeably with uptake; fidelity was often referred to as adherence; feasibility described as acceptability; and sustainability was reflected in the literature by varying, but congruent definitions, such as maintenance, incorporation, and integration [23, 75–78].

Findings from a recent review to identify instruments to measure implementation in mental healthcare included an uneven distribution of instruments across implementation outcomes [79]. Although constructs such as acceptability and adoption are well recognized among instruments, other constructs are either underdeveloped or do not lend themselves easily to instrumentation, yielding few instruments to measure a range of implementation outcomes. More groundwork is needed to enable consistency in the definition and measurement of implementation constructs rather than considering them at an abstract level. This approach will help to align areas of focus and discussions between researchers, healthcare planners, and policymakers.

### Limitations

This systematic review has several limitations. First, our research study search results may be limited given “behavioral health” or its derivatives were not used in our search string. While not all eMental healthcare studies will be indexed solely with these terms, there may be some that are. These studies would not have been included in our review. Additionally, while our search to identify unpublished research and research-in-progress was extensive, we did not search other databases, such as the NIH Reporter, which may have yielded additional eMental health technology studies. While some of the studies in the NIH Reporter may have been additionally registered in the registries we searched, some may not have been.

Another limitation is our decision to restrict inclusion of government and organizational reports from countries with known expertise and knowledge in the area of eMental healthcare. Thus, this review’s findings may not generalize to low and middle-income countries where mental healthcare systems may be organized differently and technologies used in different ways. That none of our included government/organizational documents originated in the USA, while 15 of our research studies did, could display an unrepresented sample in the review, and may explain the lack of alignment between the groups of literature we studied. Further, not all of the government and organizational websites we reviewed may have made implementation information available on the web. It was not feasible for the research team to contact all organizations by email or phone to request information that may have been in paper-based form only.

That our review was limited to English language documents also limits the literature we included in the review, and thus the generalizability of the results. We also believe that there are eMental healthcare technologies being deployed in healthcare systems that have not been scientifically investigated; thus, important implementation data for these technologies would not be available for our review.

The decision to use Proctor and colleagues’ implementation outcomes as a guiding framework was not applied a priori. Therefore, specific implementation outcome terms were not included in our search string and could have resulted in potentially relevant studies not being identified in the search results if they were indexed solely using implementation outcome terms. However, the search strategy was developed using terms specific to our population and interventions of interest. Studies of eMental healthcare technologies for children and/or adolescents are more likely to be solely indexed according to these terms and may or may not include indexing with implementation terms. Our descriptive approach to synthesizing implementation may also be criticized; however, in doing so, we believe we were able to identify an important perspective of how eMental healthcare is currently being discussed at the governmental/organizational level alongside research developments.

Finally, the inconsistent use of terminology [72–74] across the literature in this review required us to make judgment calls regarding how to categorize implementation outcomes. There also remains conceptual ambiguity and overlap among implementation outcomes (e.g., acceptability and appropriateness), which could have resulted in some factors being arguably different constructs [23, 79]. However, the difficulty in grouping these metrics within the implementation outcomes further confirms the lack of agreement regarding constructs hypothesized to affect implementation success and the identifiable measures of these constructs [80].

## Conclusion

This systematic review identified differing implementation foci between research studies and government/organizational documents, and a lack of consistent implementation taxonomies and metrics. These differences mean that the research evidence available to support eMental healthcare technologies for pediatric mental healthcare is not well aligned with the focus of governments and organizations. Using the results of this review as a guide, partnerships between researchers, healthcare planners, and policymakers may help to align implementation research, policies, and funding foci.

## Additional files

Additional file 1: Medline search strategy. (DOCX 16 kb)

Additional file 2: List of government websites and healthcare organizations searched. (DOCX 18 kb)

Additional file 3: AGREE domain scores for included government and organizational reports. (DOCX 20 kb)

Additional file 4: Quality assessment scores of research studies using the Mixed Methods Appraisal Tool (MMAT). (DOCX 54 kb)
