# Supplementary material for: Pediatric eMental healthcare technologies: a systematic review of implementation foci in research studies, and government and organizational documents
Source: Implement Sci. 2017 Jun 21;12:76. doi: 10.1186/s13012-017-0608-6 (PMC5479013; doi:10.1186/s13012-017-0608-6)
Supplement: Supplementary file 1 — Medline search strategy. (DOCX 16 kb) [file 13012_2017_608_MOESM1_ESM.docx]

**Additional File 1.** Medline search strategy.

Ovid MEDLINE(R) In-Process & Other Non-Indexed Citations and Ovid MEDLINE(R) <1973 to September 30, 2015>

1. (emental health or mmental health or e-mental health or m-mental healthor epsychiatr* or e psychiatr* or m psychiatr* or mpsychiatr* or telepsychiatr*).ti,ab,kf.

2. exp Internet/ or exp Telemedicine/

3. (chat or social media or twitter or facebook or blog*).ti,ab,kf.

4. (etherap* or e therap* or mhealth or m-health or telemedicine or telehealth or telepsychiatry or internet or social media or twitter or facebook or "mobile device*" or tablet* or smartphone* or "smart phone*").ti,ab,kf.

5. (online or web of internet).ti,kf.

6. exp Computers, Handheld/ or exp Cell Phones/ or exp Mobile Applications/

7. 2 or 3 or 4 or 5 or 6

8. exp Mental Disorders/

9. exp Mental Health/

10. ((mental or psychiatr* or mood or affective) adj2 (disorder* or disease* or illness)).ti,ab,kf.

11. mental* ill*.ti,ab,kf.

12. (bipolar or schizophrenia or anxiety disorder*).ti,ab,kf.

13. depress*.ti,kf.

14. 8 or 9 or 10 or 11 or 12 or 13

15. 7 and 14

16. 1 or 15

17. ((knowledge or research or innovation) adj3 (transfer* or translat* or utili* or uptake or implement* or adopt*)).ti,ab,kf.

18. exp Organizational Innovation/ or exp "Diffusion of Innovation"/

19. exp "Attitude of Health Personnel"/

20. (attitude* or preference* or perception* or perspective* or view*).ti,kf.

21. ((attitude* or preference* or perception* or perspective* or view*) adj3 (personnel or staff or physician* or psychiatrist* or counselor* or therapist*)).ab.

22. exp Health Knowledge, Attitudes, Practice/

23. exp Feasibility Studies/

24. exp Pilot Projects/

25. (feasibility or pilot).ti,ab,kf.

26. "Delivery of Health Care"/

27. (strategy or strategies or integrat*).ti,kf.

28. or/17-27

29. 16 and 28
