# Supplementary material for: Pediatric eMental healthcare technologies: a systematic review of implementation foci in research studies, and government and organizational documents
Source: Implement Sci. 2017 Jun 21;12:76. doi: 10.1186/s13012-017-0608-6 (PMC5479013; doi:10.1186/s13012-017-0608-6)
Supplement: Supplementary file 2 — List of government websites and healthcare organizations searched. (DOCX 18 kb) [file 13012_2017_608_MOESM2_ESM.docx]

**Additional File 2.** List of government websites and healthcare organizations searched.

**Australia**

- Government websites
  - [Department of Health](http://www.health.gov.au/)
  - [NSW Ministry of Health](http://www.health.nsw.gov.au/Pages/default.aspx)
  - [National Mental Health Commission](http://www.mentalhealthcommission.gov.au/)
  - Guidelines from relevant health organizations
  - [The Australian Council on Healthcare Standards (ACHS)](http://www.achs.org.au/)
  - [Orygen, The National Centre of Excellence in Youth Mental Health](https://www.orygen.org.au/)

**Canada**

- - Government websites
  - [Health Canada](http://www.hc-sc.gc.ca/index-eng.php)
  - [Public Health Agency of Canada](http://www.phac-aspc.gc.ca/index-eng.php)
  - [Canadian Best Practices Portal](http://cbpp-pcpe.phac-aspc.gc.ca/)
- Guidelines from relevant health organizations
  - [Canadian Institute for Health Information (CIHI)](http://www.cihi.ca/CIHI-ext-portal/internet/EN/Home/home/cihi000001)
  - [HealthCareCAN](http://www.healthcarecan.ca/)
  - [Mental Health Commission of Canada (MHCC)](http://www.mentalhealthcommission.ca/English/)
  - [Ontario Centre of Excellence for Child and Youth Mental Health](http://www.excellenceforchildandyouth.ca/)
  - [Provincial Health Services Authority](http://www.phsa.ca/)
- Others
  - [Canadian Research Index](http://www.library.ualberta.ca/databases/databaseinfo/index.cfm?ID=140)
  - [Canada Health Infoway](https://www.infoway-inforoute.ca/en/)

**Netherlands**

- Government websites
  - [European Commission](http://ec.europa.eu/index_en.htm)
- Guidelines from relevant health organizations
  - [National Institute for Public Health and the Environment](http://www.rivm.nl/en/)
  - [The Netherlands Institute for Social Research](http://www.scp.nl/english/)
  - [CIRB](https://www.cibg.nl/)
  - [Dutch Association of Mental Health and Addiction Care](http://www.ggznederland.nl/pagina/english)

**New Zealand**

- Government websites
  - [Ministry of Health NZ](http://www.health.govt.nz/)
  - [New Zealand National Health Board](http://nhb.health.govt.nz/)
- Guidelines from relevant health organizations
  - [Public Health Association of NZ inc.](http://www.pha.org.nz/)
  - [Health IT](http://www.healthit.org.nz/)
  - [Mental Health Foundation](https://www.mentalhealth.org.nz/)

**United Kingdom**

- Government websites
  - [UK Department of Health](https://www.gov.uk/government/organisations/department-of-health)
  - [National Health Service](http://www.nhs.uk/Pages/HomePage.aspx)
- Guidelines from relevant health organizations
  - [UK Public Health Associations (UKPHA)](http://www.ukpha.org.uk/)
  - [Mental Health Foundation](http://www.mentalhealth.org.uk/)
  - [National Collaborating Centre for Mental Health (NCCMH)](http://www.rcpsych.ac.uk/workinpsychiatry/nccmh.aspx)
  - [Leeds and York Partnership NHS Foundation Trust](http://www.leedsandyorkpft.nhs.uk/)

**United States**

- Government websites
  - [Health.gov](http://health.gov/)
  - [Catalog of U.S. Government Publications](http://www.gpo.gov/)
  - [National Institutes of Health](http://www.nih.gov/)
  - [Science.gov](http://www.science.gov/)
- Guidelines from relevant health organizations
  - [National Guidelines Clearinghouse (NGC)](http://www.guideline.gov/)
  - [Mental Health America](http://www.mentalhealthamerica.net/)

**All**

- Guidelines from relevant health organizations
  - [World Health Organization](http://www.who.int/en/) (WHO)
