# Supplementary material for: Pediatric eMental healthcare technologies: a systematic review of implementation foci in research studies, and government and organizational documents
Source: Implement Sci. 2017 Jun 21;12:76. doi: 10.1186/s13012-017-0608-6 (PMC5479013; doi:10.1186/s13012-017-0608-6)
Supplement: Supplementary file 3 — AGREE domain scores for included government and organizational reports. (DOCX 20 kb) [file 13012_2017_608_MOESM3_ESM.docx]

**Additional File 3.** AGREE domain scores for included government and organizational reports.

| **Author** | **Domain, %** | | | | | | **Overall Assessment, %** | **Recommend for use?** | | |
| --- | --- | --- | --- | --- | --- | --- | --- | --- | --- | --- |
|  | **Scope and Purpose** | **Stakeholder Involvement** | **Rigor of Development** | **Clarity of Presentation** | **Applicability** | **Editorial Independence** |  | **Yes** | **Yes, with modifications** | **No** |
| OCE [40] | 100 | 89 | 64 | 92 | 69 | 63 | 92 | ✓ |  |  |
| eMHA [43] | 97 | 94 | 42 | 100 | 56 | 13 | 92 | ✓ |  |  |
| Sax [38] | 97 | 25 | 59 | 83 | 75 | 42 | 83 |  | ✓ |  |
| NCCMH [39] | 100 | 69 | 86 | 83 | 48 | 83 | 83 |  | ✓ |  |
| ReachOut [37] | 97 | 44 | 48 | 58 | 56 | 21 | 83 |  | ✓ |  |
| MHN [42] | 92 | 67 | 50 | 69 | 58 | 33 | 67 |  | ✓ |  |
| DHA [44] | 100 | 100 | 32 | 50 | 46 | 21 | 58 |  | ✓ |  |
| MHCC [17] | 94 | 75 | 38 | 72 | 50 | 29 | 58 |  | ✓ |  |
| GGZ [41] | 22 | 17 | 19 | 39 | 13 | 13 | 25 |  |  | ✓ |

OCE: Ontario Centre of Excellence; eMHA: e-Mental Health Alliance; NCCMH: National Collaborating Centre for Mental Health; MHN: Mental Health Network; DHA: Department of Health and Ageing; MHCC: Mental Health Commission of Canada; GGZ: Geestelijke gezondheidszorg
