# Supplementary material for: Pediatric eMental healthcare technologies: a systematic review of implementation foci in research studies, and government and organizational documents
Source: Implement Sci. 2017 Jun 21;12:76. doi: 10.1186/s13012-017-0608-6 (PMC5479013; doi:10.1186/s13012-017-0608-6)
Supplement: Supplementary file 4 — Quality assessment scores of research studies using the Mixed Methods Appraisal Tool (MMAT). (DOCX 54 kb) [file 13012_2017_608_MOESM4_ESM.docx]

**Additional File 4.** Quality assessment scores of research studies using the Mixed Methods Appraisal Tool (MMAT).

| **Qualitative studies** | | | | | |
| --- | --- | --- | --- | --- | --- |
| **Author** | **1.1** | **1.2** | **1.3** | **1.4** | **MMAT score** |
|  | Are the sources of qualitative data relevant to address the question? | Is the process for analyzing data relevant to address the question? | Is consideration given to how findings relate to the context? | Is consideration given to how findings relate to researcher influence? |  |
| Ahmad et al. [63] | ✓ | ✓ | ✓ | ✓ | 100 |
| Pretorious et al. [59] | ✓ | ✓ | ✓ | ✓ | 100 |
| Hanley et al. [60] | ✓ | ✓ | ✓ | ✓ | 100 |
| **Quantitative randomized controlled trials** | | | | | |
| **Author** | **2.1** | **2.2** | **2.3** | **2.4** | **MMAT score** |
|  | Is there a clear description of the randomization or appropriate sequence generation? | Is there a clear description of the allocation concealment or blinding (when applicable)? | Are there complete outcome data (80% or above)? | Is there low withdrawal/drop-out (below 20%)? |  |
| Merry et al. [64] | ✓ | ✓ | ✓ | ✓ | 100 |
| Reuland et al. [48] | x | x | ✓ | ✓ | 50 |
| Gladstone et al. [49] | x | x | ✓ | ✓ | 50 |
| Eisen et al. [50] | x | x | o | o | 0 |
| **Quantitative non-randomized trials** | | | | | |
| **Author** | **3.1** | **3.2** | **3.3** | **3.4** | **MMAT score** |
|  | Are participants recruited in a way that minimizes selection bias? | Are measurements appropriate regarding the exposure / intervention, outcomes? | In the groups being compared, are the participants comparable, or do researchers take into account the difference between these groups? | Are there complete outcome data (80% or above), and, when applicable, an acceptable response rate (60% or above), or an acceptable  follow-up rate for cohort studies (depending on the duration of follow-up)? |  |
| Murphy et al. [57] | ✓ | ✓ | ✓ | ✓ | 100 |
| Branson et al. [46] | ✓ | ✓ | ✓ | ✓ | 100 |
| **Quantitative descriptive studies** | | | | | |
| **Author** | **4.1** | **4.2** | **4.3** | **4.4** | **MMAT score** |
|  | Is the sampling strategy relevant to address the research question? | Is the sample representative of the population under study? | Are measurements appropriate? | Is there an acceptable response rate (60% or above)? |  |
| Diamond et al. [54] | ✓ | ✓ | ✓ | ✓ | 100 |
| Fein et al. [55] | ✓ | ✓ | ✓ | ✓ | 100 |
| Stallard et al. [61] | ✓ | ✓ | ✓ | o | 75 |
| Horwitz et al. [58] | ✓ | ✓ | ✓ | x | 75 |
| Han et al. [53] | ✓ | ✓ | ✓ | x | 75 |

| **Mixed methods studies** | | | | | | | | | | | | | | | | |
| --- | --- | --- | --- | --- | --- | --- | --- | --- | --- | --- | --- | --- | --- | --- | --- | --- |
| **Author** | **1.1** | **1.2** | **1.3** | **1.4** | **3.1** | **3.2** | **3.3** | **3.4** | **4.1** | **4.2** | **4.3** | **4.4** | **5.1** | **5.2** | **5.3** | **MMAT score** |
|  | Are the sources of qualitative data relevant to address the question? | Is the process for analyzing data relevant to address the question? | Is consideration given to how findings relate to the context? | Is consideration given to how findings relate to researcher influence? | Are participants recruited in a way that minimizes selection bias? | Are measurements appropriate regarding the exposure / intervention, outcomes? | In the groups being compared, are the participants comparable, or do researchers take into account the difference between these groups? | Are there complete outcome data (80% or above), and, when applicable, an acceptable response rate (60% or above), or an acceptable  follow-up rate for cohort studies (depending on the duration of follow-up)? | Is the sampling strategy relevant to address the research question? | Is the sample representative of the population under study? | Are measurements appropriate? | Is there an acceptable response rate (60% or above)? | Is the design relevant to address the question? | Is the integration of data / results relevant to address the question? | Is consideration given to the limitations associated with this integration in a triangulation design? |  |
| Gonzales et al. [45] | ✓ | ✓ | ✓ | ✓ |  |  |  |  | ✓ | ✓ | ✓ | ✓ | ✓ | ✓ | ✓ | 100 |
| Hetrick et al. [62] | ✓ | ✓ | x | ✓ |  |  |  |  | ✓ | ✓ | ✓ | ✓ | ✓ | ✓ | ✓ | 100 |
| Fothergill et al. [52] | ✓ | ✓ | ✓ | ✓ |  |  |  |  | ✓ | ✓ | ✓ | ✓ | ✓ | ✓ | ✓ | 100 |
| John et al. [47] | ✓ | ✓ | x | ✓ | ✓ | ✓ | ✓ | x |  |  |  |  | ✓ | ✓ | ✓ | 75 |
| Iloabachie et al. [51] | ✓ | ✓ | ✓ | ✓ |  |  |  |  | ✓ | ✓ | ✓ | x | ✓ | ✓ | ✓ | 75 |
| Salloum et al. [56] | ✓ | ✓ | ✓ | x |  |  |  |  | ✓ | ✓ | ✓ | ✓ | ✓ | ✓ | ✓ | 75 |

✓=yes; x=no; o=can’t tell; RCT: Randomized Controlled Trial; MMAT=Mixed Methods Appraisal Tool; All studies undergoing quality assessment passed the screening questions: 1) Clear research question? 2) Do collected data address the question?
